# Dances as Windows into Insect Perception

**DOI:** 10.1371/journal.pbio.0020216

**Published:** 2004-07-13

**Authors:** Lars Chittka

## Abstract

Honeybees signal the location of food sources to their hive- mates using a "dancing" flight pattern. Translating these patterns, scientists learn what bees perceive

Experimental psychologists working with humans have a fundamental advantage over scientists studying the behaviour of other animals. This is because human subjects can give a verbal account of their experience. For example, they can report: ‘These two lights of different colour look equally bright’ or ‘This object looks further away than that one’. Such direct reports facilitate studying how information from the sensory periphery, that is, the sense organs that actually interface with the environment, is processed in the brain.

The perceptual world of animals is often very different from that of humans. Many animals have sensory facilities that we humans lack; for example, insects can see ultraviolet and polarised light. But how they actually perceive the world, based on information from their sensory periphery, is often beyond our grasp. Because animals cannot describe their sensations, our access to them is often based on indirect psychophysical tests, where animal performance depends fundamentally on motivation and training method (Chittka et al. 2003). However, some animals do in fact describe the world around them, but not necessarily in ways that we might intuitively understand. Perhaps the best example of this are the honeybees (genus Apis), which have a symbolic ‘language’ that nestmates use to communicate with each other about profitable food sources. By eavesdropping on this communication, scientists have recently obtained a unique perspective into the perceptual world of insects.

How does the dance language work? A triumphant scout bee returns from the field, and advertises the location of a newly discovered food source to nestmates. To do this, the forager performs a repetitive sequence of movements, the so-called waggle dance, which is one of the most intriguing examples of complex animal behaviour. The successful forager wiggles her abdomen provocatively from side to side, moving forward in a straight line. Then she runs in a half circle to the left, back to her starting point, performs another straight wiggle run along the path of her first, and then circles to the right (Figure 1). This pattern is repeated multiple times, and is eagerly attended by unemployed bees in the hive. Shortly after such dances commence, dozens of newly recruited foragers arrive at the food source being advertised.

**Figure 1:**
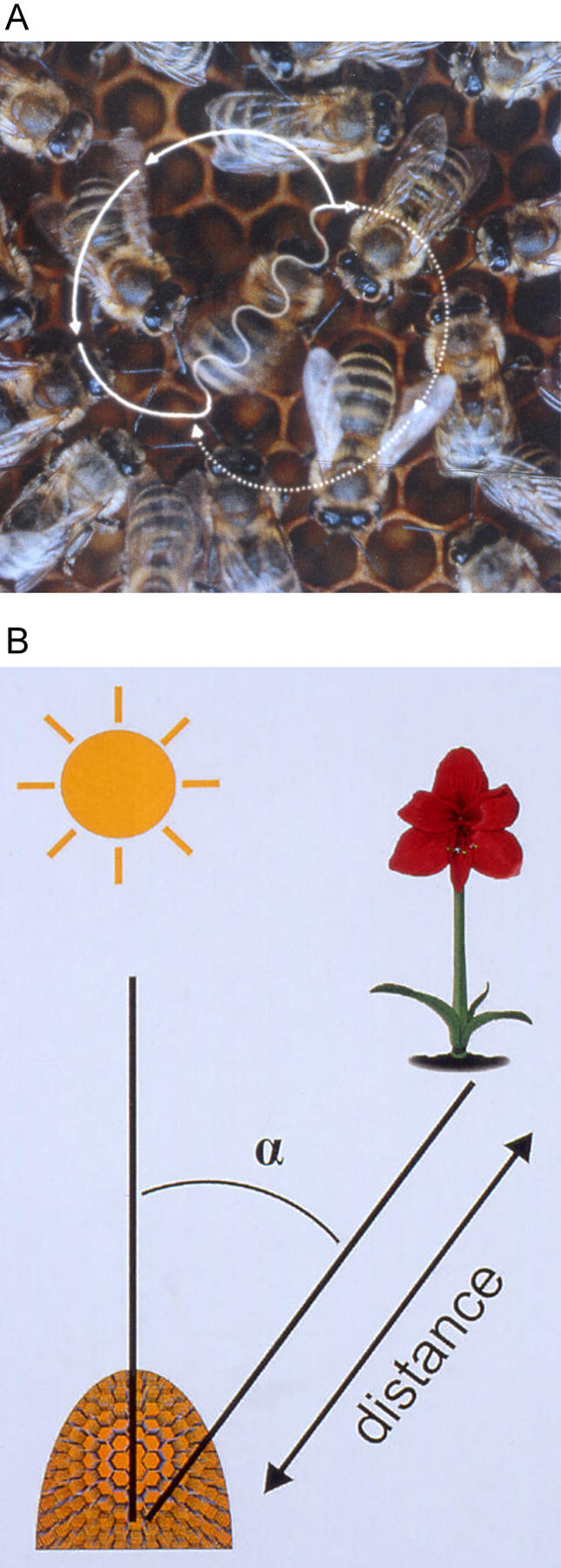
Figure-Eight-Shaped Waggle Dance of the Honeybee (Apis mellifera) A waggle run oriented 45° to the right of ‘up’ on the vertical comb (A) indicates a food source 45° to the right of the direction of the sun outside the hive (B). The abdomen of the dancer appears blurred because of the rapid motion from side to side. (Figure design: J. Tautz and M. Kleinhenz, Beegroup Würzburg.)

In the 1940s, Nobel laureate Karl von Frisch deciphered the code hidden in this seemingly senseless choreography performed on vertical honeycombs in the darkness of the hive (reviewed in von Frisch 1967). He found that the angle of the waggle run from the vertical is equal to the angle between the sun's azimuth and the indicated food source outside the hive. For example, if a food source is found in the direction of the sun, the dancer will waggle ‘straight up’ the vertical comb. If food is found 45° to the right of the sun's direction, the waggle run will be oriented 45° to the right of vertical on the comb (Figure 1). The distance to the target, a flower patch with abundant nectar or pollen, is encoded in the duration of the waggle run: the longer the bee waggles, the larger the distance of the food from the hive. No other species (besides humans) uses a similarly symbolic representation to communicate information from the real world.

But how do bees measure the flight distance that they communicate so precisely? It was previously thought they do this by measuring the energy used as they fly (Heran 1956). However, doubts emerged when it was found that distance estimation by bees could be manipulated by altering the number of landmarks between the hive and a food source, suggesting bees were counting landmarks encountered en route (Chittka and Geiger 1995). In an elegant experiment, Esch and Burns (1995) tapped into the bees' dance language to access their subjective assessment of flight distance. They let bees forage from a food source 70 m from the hive and recorded the dance distance code of the returning foragers. Subsequently, the feeder was attached to a weather balloon, and slowly lifted to an altitude of 90 m—so that the distance between the hive and the food now increased from 70 m to 114 m. Correspondingly, foragers should have indicated a longer distance, by stretching their waggle run duration. But, in fact, the perceived distance (as indicated in the dance) *decreased* by more than 50%! This clearly shows that bee perception of distance cannot solely be based on energy expenditure, since a longer flight that cost more energy was danced as a shorter ‘distance’ in the waggle run.

So what actually drives the bee odometer? Because the landscape bees pass in flight moves more slowly when bees fly at higher altitudes, Esch and Burns (1995) conjectured that foragers process the speed with which visual contours move across the eye (optic flow), and integrate this with travel time. To confirm this hypothesis, Srinivasan et al. (2000) further exaggerated the experienced image flow, by training bees to fly through narrow chequered tunnels. These bees grossly overestimated actual travel distance, bragging to their nestmates that they had flown 195 m when in fact they had flown 6 m. Attendees of these dances promptly believed the high-class swindle, and searched for food at remote locations that the dancers had never even visited (Esch et al. 2001).

The quality of information available about the velocity of the passing landscape will depend, of course, on the sensitivity of the eyes. The eyes of bees contain three types of colour receptors, with maximum sensitivity in the ultraviolet, blue, and green domains of the spectrum (Autrum and von Zwehl 1964). Their excellent colour vision is optimal for flower identification (Chittka 1996), but do they also use it to measure the image velocity of the passing landscape? Surprisingly, the answer is no—bee odometry is in fact totally colour blind. Chittka and Tautz (2003) found that bees use exclusively the signal from their green receptors for measuring image velocity (Figure 2), confirming earlier reports that motion vision in bees is mediated only by this receptor type (Giurfa and Lehrer 2001; Spaethe et al. 2001). Thus, the level of intensity contrast present in the scene strongly influences the bees' subjective experience of flight distance (Chittka and Tautz 2003; Si et al. 2003).

**Figure 2:**
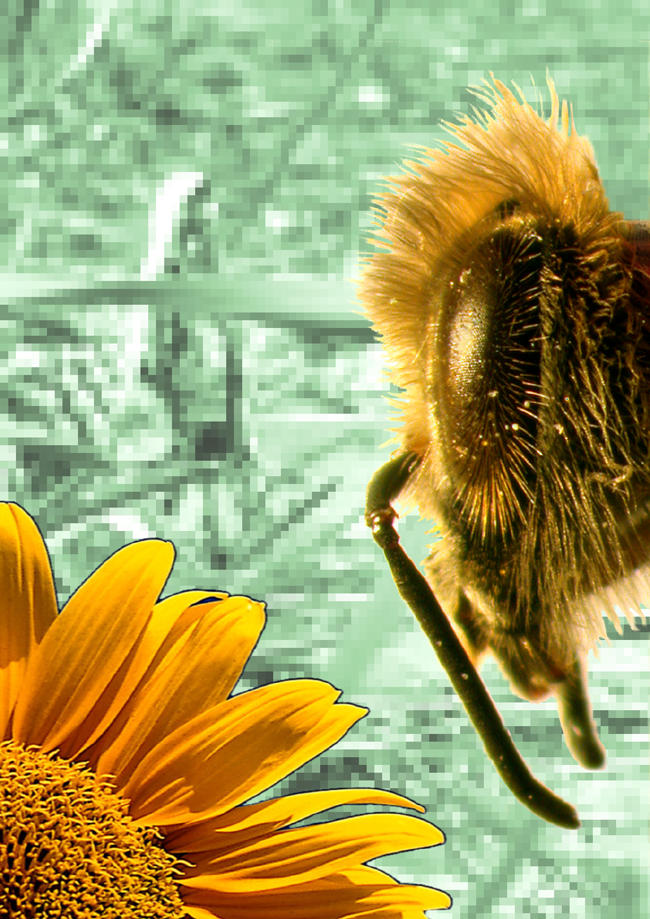
Bees Use Different Visual Cues When Viewing Flowers and Landscape Image Motion Although bees see flowers in colour, they do not analyse the colours of the landscape image that moves across the eye as they fly. Their perception of landscape motion is colour-blind; motion vision is driven solely by a single spectral receptor type, the bees' green receptor. This is reflected in the distance code of the dance: the more green contrast is present in the scene, the further bees ‘think’ they have flown. (Figure design: F. Bock, Beegroup Würzburg.)

With so many external variables influencing distance estimation, it seems unlikely that the honeybee odometer would be very robust in natural conditions. Now, as reported in this issue of *PLoS Biology*, Tautz et al. (2004) have quantified the bees' subjective experience of distance travelled when they fly over natural terrain with varying levels of contrast. Specifically, they compared the dances of bees flying over water (scenery with low visual contrast) with those of bees flying over land (scenery with relatively high contrast). They trained bees to forage at a feeder on a boat, which was paddled increasing distances from the hive, until it reached an island. All the while, observers at the hive deciphered the dances of the bees returning from the feeder. Interestingly, bees flying 200 m over water hardly appeared to register an increase in travel distance, whereas the same increase in distance flown over land resulted in a substantial increase in perceived flight distance. This is consistent with the hypothesis that the bees' odometer is largely based on visual, external cues and demonstrates that this system is sensitive to visual contrast.

But there must be something else beside visual cues. Navigation over water, in the near absence of visible ground features, is extremely difficult without a reliable internal instrument measuring travel speed. This is the case even for us humans with sophisticated measuring devices: malfunctioning air speed indicators have been responsible for several airplane crashes into water, for example Birgenair Flight 301 and AeroPeru Flight 603 in 1996. Heran and Lindauer (1963) likewise observed that honeybees flying over lakes sometimes lost altitude and plunged into the water. However, the new study by Tautz et al. (2004) also shows that most bees will reliably fly over prolonged stretches of water without accident. Furthermore, even though bees experience only a small increase in subjective travel distance when flying over water, it is not zero. This indicates that bees do perhaps resort to an internal measure of flight distance when other cues fail. For example, bumblebees walking to a food source in absolute darkness, that is, in the complete absence of visual cues, are able to correctly gauge travel distance (Chittka et al. 1999), indicating that an internal odometer, possibly based on energy consumption, also exists. It appears that animal navigation, just like aviation, relies on multiple backup systems that support each other and can compensate if one system fails in a certain context.

Spying on honeybee dances can not only tell us about the cues they use for navigation, but also allows insights into the cognitive architecture that governs other aspects of bee behaviour, such as the assessment of flower quality. We've learned that bees prefer high over low nectar concentrations because this is reflected in their dances. When bees find better nectar, they dance more enthusiastically, that is, the number of dance circuits per minute increases (Seeley et al. 2000; Waddington 2001). However, Waddington (2001) found that the relationship between actual and perceived nectar quality is nonlinear. In fact, it is a positive but decelerating relationship, so that an increase in sucrose concentration from 10% to 20% results in twice the difference in dance rate that an increase from 50% to 60% does. Interestingly, the perceived change in quality is stronger when there is a *decrease* than when there is an *increase* in nectar quality of the same magnitude. Such asymmetric perception of gains and losses is well known in humans, where it has been linked to risk-aversive behaviour (Tversky and Kahnemann 1981). Unfortunately, animal subjects often do not yield this type of information very readily. Only in their own language do they reveal many of their perceptual peculiarities. Using the bee language as a window into insect visual perception has been a wonderful tool and is a promising avenue for further research into the question of how miniature brains encode the world around them.
